# Applying “Consensus Design” in the Development of Psychiatric Facilities

**DOI:** 10.1192/j.eurpsy.2023.173

**Published:** 2023-07-19

**Authors:** M. Voss

**Affiliations:** Department of Psychiatry and Psychotherapy, Charité University Medicine & St. Hedwig Hospital, Berlin, Germany

## Abstract

**Abstract:**

I will point out the important role of a thorough planning process in which all stakeholders work together starting in early phases of the design process („phase 0“) and engage in a truly interdisciplinary and iterative process throughout the entire planning phase as well as the building phase (where often *ad hoc* decisions have to be made in order to adjust to unforeseen circumstances).

I will examine the terms *"Consensus Design"* and *"Evidence-Based Design"* and relate them to lived reality by giving a number of examples from own experience. Here I will contrast different approaches in carrying out the planning process and demonstrate how only a truly interdisciplinary and iterative process can result in individualised and optimised therapeutic environments, strengthen identity and reduce stigmatisation.

As a support to future projects which workshop participants may be involved in, I will share some of the basic methods and tools which I have seen or used to help build and maintain this type of collaborative conversations throughout project phases.

**Disclosure of Interest:**

None Declared

